# Exercise-Induced Asthma in Asthmatic Children of Southern Iran

**DOI:** 10.5539/gjhs.v7n2p115

**Published:** 2014-09-28

**Authors:** Abbas Fayezi, Reza Amin, Sara Kashef, Soheila Al Yasin, Mohammad Bahadoram

**Affiliations:** 1 Department of Allergy and Clinical Immunology, School of medicine, Ahvaz University of Medical Sciences, Ahwaz, Iran; 2 Department of Allergy and Clinical Immunology, Nemazee Hospital, Shiraz University of Medical Sciences, Shiraz, Iran; 3 Departement of Allergy and Clinical Immunology, Allergy Research Center, Shiraz University of Medical Sciences, Shiraz, Iran; 4 Medical Student Research Committee, Ahvaz Jundishapur University of Medical Sciences

**Keywords:** asthma, exercise challenge, exercise-induced asthma

## Abstract

**Background:**

Asthma is a common illness, especially among children. Exercise-induced asthma is an important consideration, both as a factor, limiting physical activity of patients, and also as an indicator of poor long term control. We investigated pre-Valence of exercise-induced asthma in a group of asthmatic children living in southern Iran.

**Methods:**

We conducted treadmill exercise challenge test in 40 young asthmatic patients aged 6 to 18. After 8 minutes exercise to achieve 80% of maximum heart rate predicted for age, patients were examined and spirometry values recorded at frequent intervals. We defined exercise-induced asthma as 10% or more decline in Forced Expiratory Volume in one second (FEV1) within 30 minutes after exercise challenge.

**Results:**

Of 40 patients evaluated, 22 patients (55% of total) met our criteria for exercise-induced asthma. Most positive responses (7 of 22, 31.8%) occurred at about 10 minutes after exercise. Cough was the most consistent sign (18 of 22 patients, 81%). In 2 patients (9%), FEV 1 decline did not accompany any symptom or sign.

**Conclusion:**

We concluded that Exercise- induced asthma occurs in a relatively smaller subset of southern Iranian asthmatic children. Also treadmill exercise challenge performed by a trained staff, following standard protocol and using enough monitoring and precautions is safe and diagnostic in children and adolescents.

## 1. Introduction

Exercise-induced asthma (EIA) is defined as the condition in which exercise induces symptoms in patients who have asthma (Weiler et al., 2007). It is also defined as a syndrome of cough and/or wheezing, or symptoms of chest tightness or pain associated with 6-8 minutes of continuous and strenuous exercise in individuals with known asthma (Randolph, 2008). Exercise- induced bronchoconstriction in non-asthmatic athletes is a distinct entity not considered here. Asthma symptoms with exercise is considered by some authors as a measure of control, or as a manifest-tation of chronic asthma rather than a distinct entity (Karjalainen et al., 2000; Verges, Flore, Blanchi, & Wuyam, 2004; Helenius, Lumme, & Haahtela, 2005).

An estimated 12% of the pediatric population has exercise induced bronchoconstriction and 30% of these patients may develop adult asthma (Porsbjerg et al., 2005). Up to 90% of asthmatic patients are reported to have EIA (Weiler et al., 2007; Randolph, 2008). Asthma is a disease of genetically predisposed persons exposed to certain environmental stimuli, so ethnic variability may play a role in some aspects of disease expression. Few studies are available estimating EIA in Iranian asthmatic children. The burden of the disease has not been determined in terms of direct and indirect costs in this country. In a recently published data, in a population of school adolescent girls, labeled as to have asthma on the basis of the response to a questionnaire, and using free running to provoke symptoms, EIA was estimated to be present in about 60 percent of symptomatic participants (Marefati, Nikbine, & Boskabady, 2011). The objective of this study is to estimate prevalence of EIA in a population of asthmatic children living in southern Iran, diagnosed and followed by university allergists, using standard treadmill exercise challenge test to provoke symptoms.

## 2. Methods

### 2.1 Study Population

Among young patients 6 to18 years old with known asthma, coming to Motahari Pediatric Asthma Clinic to follow, 50 were enrolled in the study over a period of about one year, an acceptable sample size considering time consuming protocol and some difficulty in convincing parents to take part in the study. Patients were selected consecutively with different disease severity and different steps of treatment, but they were not categorized on the basis of taking or not any medication. Patients were planned to be excluded if unable to perform pulmonary function test (PFT), had FEV1 below 65% predicted, or had any concomitant disease or disability.

### 2.2 Study Intervention

Exercise testing was done by 40 patients. 4 patients were unable to perform pulmonary function test and 6 patients had baseline FEV1 below 65% predicted and therefore excluded. PFT was done by Jaeger (Erich Jaeger GmbH &co-KG) spirometer.

Patient’s age, gender, pre-exercise dyspnea, cough and wheezing if present, temperature and humidity were recorded. Exercise test was conducted after a baseline PFT measurement, using treadmill, adjusting the speed to keep heart rate at about 80% of maximum estimated for age. Predicted maximum heart rate is calculated as: 220 – age in years (Crapo et al., 2000). After 6 minutes of running at desired heart rate, under heart rate and oxygen saturation monitoring, patients were undergone serial PFT measurements at 2.5, 5, 10, 15, 20, and 30 minutes after exercise. Exercise challenge was considered positive if FEV1 dropped 10% or more comparing baseline values according to the definition supported by most experts (Anderson & Kippelen, 2005; Helenius, Lumme & Haahtela, 2005; Parsons, & Mastronarde, 2005; Anderson 2006).

All challenges were done in humidity of 50% or less and a maximum temperature of 26 degree Celsius, that is an optimal condition to perform the test according to recommendations made by American Thoracic Society (Karjalainen et al., 2000).

At each measurement, other parameters such as Peak Expiratory Flow (PEF) and Forced Expiratory Flow between 25% to 75% of expiratory time (FEF 25-75) were also measured and changes compared to baseline. Also O2 saturation was recorded and patients were examined to see if dyspnea, cough, and wheezing develop.

Patients who developed symptoms and suffered decreases in lung function and met our criteria of EIA, were treated by bronchodilator and received recommendations about maintenance therapy if indicated, then discharged after improvement and a period of observation.

### 2.3 Statistical Analysis

Statistical analysis was done using Paired T-test and Chi-square test. Paired T-test was used to analyse changes in clinical findings and spirometric values before and after exercise. Chi-square test was used to analyse interrelation between changing parameters and distribution of changes over time and among different genders.

The protocol for this research project has been approved by the Ethic Committee of the Shiraz University of Medical Sciences and it conforms to the provisions of the Declaration of Helsinki in 1995 (as revised in Edinburgh, 2000).

## 3. Results

Of 50 patients entering the study, 10 were excluded, 4 patients were unable to perform PFT and 6 patients had baseline FEV1 below 65% predicted and therefore excluded. Of 40 patients undergone exercise testing, 22 patients (55% of total) met our criteria for EIA, defined as 10% or more decline in FEV1 after exercise. Most positive responses occurred at about 10 minutes post-exercise (7 of 22 patients, 31.8% of positive responses). Cough was the most consistent sign (in 18 of 22 patients, 81% of positive responses) followed by wheezing (in 14 patients) and dyspnea (in 13 patients) in decreasing order of frequency. While none of patients showed decline in O_2_ saturation. In two patients FEV1 reduction did not accompany any symptom or sign. No one of patients suffered oxygen saturation reduction after exercise. In 19 of 22 patients with positive response (86.4%), PEF decline was more than 10% and in 15 patients of 22 positive responses (68.2%), FEF25-75 decline was more than 25%. Gender had no statistically meaningful effect on development of EIA. Of 22 patients with positive responses 16 were male (72.7%) that was proportional to 31 of total 40 patients (77.5%).

## 4. Discussion

Physical exertion is one of many non-pharmacologic and non-immunologic stimuli that can produce episodes of airway obstruction in patients with asthma. Physical activity is the second leading cause of acute airway obstruction and ranks only behind viral upper respiratory tract infections in this regard (Parsons et al., 2013). In fact, most if not all patients with asthma develop symptoms of asthma after a suitable exercise challenge (Crapo et al., 2000). The term exercise-induced bronchoconstriction (EIB) is used to describe the airway obstruction that occurs in association with exercise without regard to the presence of chronic asthma (Weiler et al., 2007).

Exercise-induced asthma (EIA) is conventionally defined as at least 10% or more decline in FEV1 after exercise (Anderson & Kippelen, 2005; Helenius, Lumme, & Haahtela, 2005; Parsons, & Mastronarde, 2005; Anderson 2006). Some authors consider 15% or more decline in FEV1 as the cutoff point to diagnose exercise induced asthma (Parsons et al., 2013). In this study, we defined exercise-induced asthma as 10% or more decline in FEV1 after exercise. A 10% or more decline in PEF or at-least 25% reduction in FEF25-75 may also be considered as consistent to EIA (Bacharier & Guilbert, 2012). The exercise to provoke these changes may be running on a treadmill at a defined intensity, duration and in a dry and relatively cold environment, or the exact venue in which exercise elicits symptoms. As noted earlier 55% of recruited patients in our study developed EIA; this is in contrast to latest reported rates of up to 90% in other parts of the world (Weiler et al., 2007; Randolph 2008) and some what close to recent report of the condition in 60 percent of symptomatic asthmatic patients in a population of school adolescent girls in Iran (Marefati & Nikbine, 2011). The relatively low rate of EIA in our study (55%) may be due to inhaled corticosteroid (ICS) therapy in most patients, as they were known asthmatics and under follow up of university allergists, or possibly ethnic differences. Two patients had FEV1 decline in the absence of any symptom or sign, emphasizing the importance of an objective provocation test to be considered in the long term follow up of patients.

We found baseline PFT to be a poor predictor of EIA, as significant FEV1 decline occurred in some patients with normal baseline lung function. In two studies (Rupp, Guill, & Brudno, 1992; Rupp, Brudno, & Guill, 1993) the same results have been shown in athletes. We also found no significant decline in PEF in about 14% of patients with EIA. Use of PEF for this purpose is not recommended (Weiler et al., 2007). One limitation in our study was that patients were not categorized on the basis of taking or not controller medications, which certainly could affect the response to exercise challenge.

The most common problem encountered in exercising a patient with asthma is severe bronchoconstriction. ECG abnormalities, falling blood pressure, and severe decrease in O2 saturation are among other adverse events that need to be watched for (Crapo et al., 2000). No adverse event occurred in patients undergone the procedure in our study and we found the test to be safe if conducted as we described.

Finally, exercise challenge is time consuming and needs enough space and equipment which make it somewhat difficult to perform in outpatient clinics.

## 5. Conclusion

Our study suggests EIB occurs in a relatively smaller subset of asthmatic children in southern Iran. Also treadmill exercise challenge test done by an experienced technician, following standard protocols with enough monitoring and precautions is safe in children and adolescents.
